# Segmentation of Laser Marks of Diabetic Retinopathy in the Fundus Photographs Using Lightweight U-Net

**DOI:** 10.1155/2021/8766517

**Published:** 2021-10-19

**Authors:** Yukang Jiang, Jianying Pan, Ming Yuan, Yanhe Shen, Jin Zhu, Yishen Wang, Yewei Li, Ke Zhang, Qingyun Yu, Huirui Xie, Huiting Li, Xueqin Wang, Yan Luo

**Affiliations:** ^1^State Key Laboratory of Ophthalmology, Image Reading Center, Zhongshan Ophthalmic Center, Sun Yat-Sen University, Guangzhou 510060, China; ^2^Department of Statistical Science, School of Mathematics, Southern China Research Center of Statistical Science, Sun Yat-Sen University, Guangzhou 510275, China; ^3^Department of Statistics and Finance, School of Management, University of Science and Technology of China, Hefei, Anhui 230026, China; ^4^Xinhua College, Sun Yat-Sen University, Guangzhou 510520, China

## Abstract

Diabetic retinopathy (DR) is a prevalent vision-threatening disease worldwide. Laser marks are the scars left after panretinal photocoagulation, a treatment to prevent patients with severe DR from losing vision. In this study, we develop a deep learning algorithm based on the lightweight U-Net to segment laser marks from the color fundus photos, which could help indicate a stage or providing valuable auxiliary information for the care of DR patients. We prepared our training and testing data, manually annotated by trained and experienced graders from Image Reading Center, Zhongshan Ophthalmic Center, publicly available to fill the vacancy of public image datasets dedicated to the segmentation of laser marks. The lightweight U-Net, along with two postprocessing procedures, achieved an AUC of 0.9824, an optimal sensitivity of 94.16%, and an optimal specificity of 92.82% on the segmentation of laser marks in fundus photographs. With accurate segmentation and high numeric metrics, the lightweight U-Net method showed its reliable performance in automatically segmenting laser marks in fundus photographs, which could help the AI assist the diagnosis of DR in the severe stage.

## 1. Introduction

Diabetic retinopathy (DR) is one of the most common complications of diabetes and the leading cause of irreversible visual loss globally [1]. For patients who have developed severe DR, panretinal photocoagulation (PRP) is one of the main treatments to reduce the risk of blindness. Laser marks are the scars left after retinal laser treatments. Identifying the position of laser marks on fundus photographs provides information of the received retinal laser treatment and thus is significant for the assistance of R3A and R3S DR stage grading in the Diabetic Eye Screening Guidance of the National Health Service (NHS) in the United Kingdom (UK) [2]. The R3A is classified as active proliferative DR (PDR) with at least one of the following active proliferate DR characteristics: new vessels on disc, new vessels elsewhere, preretinal or vitreous hemorrhage, and preretinal fibrosis with or without tractional detachment. The R3S is classified as the a stable stage of PDR after panretinal laser treatment and can present stable preretinal fibrosis, but without any other active proliferate DR characteristics [3]. The stable R3S status with panretinal laser marks can be distinguished from an active R3A, and the treatments of these two stages are different [2]. Laser marks appearing on nasal proximity to optic disc no closer than 500 microns, on temporal proximity to macular center no closer than 3000 microns, and at the superior/inferior limit that no further posterior than 1 burn within the temporal arcades, are believed to be the signal of receiving PRP [4]. Patients diagnosed as R3S could be monitored in the annual screen, while the active treatments such as PRP or intravitreal injection of anti-VEGF drug should be received by patients diagnosed as R3A [2]. Moreover, determining the position of laser marks is of great importance to patients with DR for the follow-up treatment. For instance, many patients may not complete all laser photocoagulation at one treatment and need to receive laser photocoagulation several times because of the progress of their disease or personal tolerance. Besides, if patients are detected new active proliferate lesions on the retina even with the presence of laser marks, their DR gradings may revert to R3A, and the patients may be urged to receive active treatments [2, 5]. In automatic retinal diagnostic systems, the existence of laser marks on the fundus photographs may hinder the further retinal image assessment [6]. Hence, segmenting laser marks in fundus photograph images becomes clinically important.

Traditional detections [6–8] and deep learning methods [9] have put effort into detecting the existence of laser marks. However, they do not give enough attention to the exact locations of laser marks on the fundus photograph images. The traditional methods, which make full use of morphological characteristics related to laser marks for detection, in a sense offer the candidate regions of laser marks, but the extraction of candidate regions tends to be relatively poor with irregular boundaries. The deep learning method, which is a state-of-the-art machine learning technique that has shown its superiority in the diagnosis of some diseases [10–12], especially the DR screening in the field of ophthalmology [13–16], extracts features directly from images for detection and attains relatively high detective accuracy, but it does not provide interpretable explanations for its decisions [17, 18]. Also, it has long been a challenge to include medical knowledge within machine learning algorithms, especially within the deep learning algorithms [11, 18, 19]. Deficient attention to segmenting laser marks in fundus images may also in part result in difficulty attaining image data accurately annotated by well-trained ophthalmologists. So far, there are no public fundus photograph datasets annotating laser marks pixel by pixel. Thus, few studies have been involved in the segmentation of laser marks in fundus photograph images.

In this study, a deep learning method, the light version of U-Net [20], named lightweight U-Net, was adapted to segment laser marks in fundus photographs using the dataset we proposed. The lightweight U-Net inputted a fundus image and outputted the probability map localizing the potential area of laser marks. Furthermore, to meliorate the segmentation maps, we introduced two postprocessing procedures developed from clinical practice. These procedures further improved the segmentation accuracy of the prediction. The well-performed model would be of great help for ophthalmologists serving as an essential component of the DR computer-aided diagnosis system.

## 2. Materials and Methods

### 2.1. Datasets

The fundus photographs with laser marks in this dataset were RGB images in a JPG or JPEG format and obtained from the Image Reading Center of Zhongshan Ophthalmic Center, Sun Yat-Sen University, China. The fundus photographs were collected from the clinical department of Zhongshan Ophthalmic Center, or the DR screening charity project of Lifeline Express in China, or the internet public data. A set of 154 fundus photographs with laser marks comprised of two subdatasets: one contains 84 images manually segmented by experienced graders at the Image Reading Center only once, and the other contains the rest 70 images manually and independently segmented by three experienced graders at the Image Reading Center. Since the manual segmentation of 84 images in the first dataset was relatively coarse, which might lead to a less precise estimation, we only used it in the pretraining session.

For the second dataset containing 70 images, the three experienced graders were asked to mark all the pixels ensured as laser marks. The gold standards of the images were labeled by at least two graders. Since this subdataset had more precise manual segmentations, it was used to construct the formal data set. These 70 fundus photographs with gold standards were randomly divided into the formal training set with 50 images and the testing set with 20 images, respectively.

In our dataset, the microaneurysms, retinal hemorrhages, hard exudates, soft exudates, venous beading, intraretinal microvascular abnormality, NVE/NVD, fibrous proliferation, preretinal hemorrhage, vitreous hemorrhage, and tractional retinal detachment were detected in about 95.71%, 98.57%, 70.00%, 12.86%, 0.00%, 11.43%, 5.71%, 2.86%, 1.43%, 0.00%, and 0.00% of the 70 images, respectively. Besides, among all 154 fundus photographs, 75.97% are at the R3S stage, 3.25% are at the R3A stage, and the remaining 20.78% are photos that are at other stages. And 51.30% of the images are with PRP, and 42.21% are with partial retinal laser marks. All of the laser marks in our data were at the late stage.

It should be noted that every image collected in the dataset was used to make clinical diagnoses, leading to the characteristic inconsistency of these images because photos were acquired by more than one specific camera model, such as CIRRUS, Cobra, and Canon. Shot by various types of cameras, the images had different resolutions (ranging from 1116 × 1080 pixels to 4928 × 3264 pixels), fields-of-views (from 45 to 60 degrees), hues (whitish, yellowish, reddish, etc.), centers of the fundus images (either macula lutea or optic disc (OD), pupil diameters, and so on. All these variations contributed to the diversity of our dataset, enabling a more generalized and robust deep learning algorithm for laser marks segmentation.

### 2.2. Image Preprocessing and Augmentation

It was essential to preprocess the images to summarize the commonness artificially because fundus photographs in the image dataset varied in size, resolution, and hue due to the multiplicity of camera devices. Preprocessing helped diminishing differences in the intrinsic feature distributions.

First, images were all resized to 512 × 512 pixels. The next three steps were successively implemented on each channel to eliminate the outputted overall tone brightness variance among images. The *Z*-score standardization, resulting in an image with a mean of 0 and a variance of 1, was calculated using the following formula:

(1) $$\begin{array}{c} z_{ijk}=\frac{x_{ijk}-\mu_{jk}}{\sigma_{jk}},\\ i=1,2,\cdots ,N^{\left(\text{img}\right)},j=1,2,\cdots ,p,k=1,2,3, \end{array}$$

where *N*^(img)^ is the total number of images in the training set, *p* is the number of pixels in a channel of an image (for a 512 × 512 image, the number of pixels *p* is 262144), and *k* = 1, 2, 3 represents the three channels (red, green, blue) of an image. And *x*_*ijk*_ represents the *j*th pixel value in the *k*th channel of the *i*th image, *μ*_*jk*_ = ∑_*i*_*x*_*ijk*_/*N*^(img)^, and $\sigma_{jk}=\sqrt{\sum_{i}{\left(x_{ijk}-\mu_{jk}\right)}^{2}/N^{\left(\text{img}\right)}}$ is the mean and the standard deviation of the corresponding pixel points of all images in the training set, respectively. The outcome *z*_*ijk*_ is the adjusted value of that specific pixel.

Inner image minimum-maximum normalization then followed, aiming to rescale the gray values into a scale of 0 to 255. This process was enabled by

(2) $$\begin{array}{c} v_{ijk}=\frac{z_{ijk}-\underset{j=1,\cdots ,p}{\min}\left(z_{ijk}\right)}{\underset{j=1,\cdots ,\text{p}}{\max}\left(z_{ijk}\right)-\underset{j=1,\cdots ,p}{\min}\left(z_{ijk}\right)}\times 255,\\ i=1,\cdots ,N^{\left(\text{img}\right)},j=1,\cdots ,p,k=1,2,3, \end{array}$$

where *z*_*ijk*_ is obtained in the previous step, and $\underset{j=1,\cdots ,p}{\min}\left(z_{ijk}\right)$ and $\underset{j=1,\cdots ,p}{\max}\left(z_{ijk}\right)$ stand for the minimum and maximum value of *z*_*ijk*_ in the *k*th channel of *i*th image, respectively. And *v*_*ijk*_ is the final value restricted in the range between 0 and 255.

**Figure 1:**
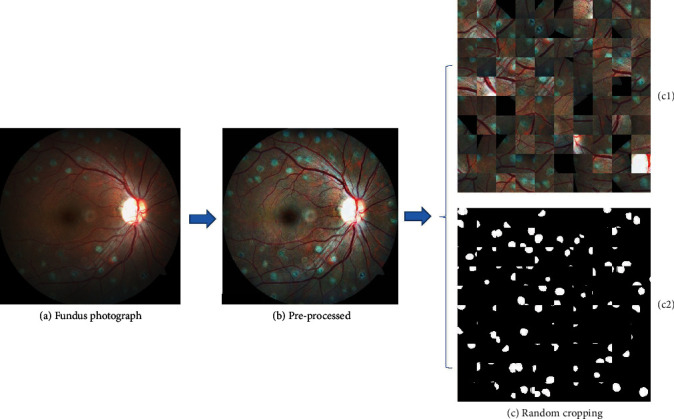
Presentation of the preprocessing and data augmentation session. (a) A sample from the training set. (b) The matching preprocessed image. (c) The examples of the randomly cropped patches (c1) and the corresponding labeled patches (c2).

The contrast limited adaptive histogram equalization (CLAHE) [21] and the gamma correction (*γ* = 1/1.2) [22] were conducted sequentially to enhance the contrast in the image. The grayscale values were then divided by 255 to transform them back to the 0 and 1 range. By performing these preprocessing steps on all three channels separately and then combining them back together, the contrast could be effectively enhanced between the laser marks and background while feature discrepancies caused by camera models would be weakened (Figure 1).

The random cropping technique was applied to effectually augment the training set due to data scarcity [23]. A total of 500,000 patches were extracted from the formal training set that contained 50 fundus images. Completely black patches extracted from the peripheral black area that contained no information would be excluded. As a result, from each training image, 10,000 randomly centered patches with 48 × 48 pixels in size were extracted. This size was chosen because the patches of the size were able to determine whether there were one or two laser marks in the images, thus enabling the network to learn the characteristics of these specific lesions. The corresponding ground truth label patches were also extracted (Figure 1) to match the augmented training set. In the testing process, we cropped the images into pieces 48 × 48 pixels in size, a coherent size to the training ones. Unlike the training set where the central locations were randomly chosen, a sliding operation with stride 5 (five pixels were moved each time) was adopted in the testing set to clip the patches. Although some parts of the original images were repeatedly seized, i.e., the patches overlapped, it benefitted the accuracy of the prediction, as a single-pixel might be predicted several times. The patches cropped at the right or lower margins might not be of size 48 × 48 because of the sliding operation; as to these images of incompatible sizes, we used a zero padding strategy on that specific margin (or those margins) to preserve the marginal information. In our case, a total of 3364 patches could be attained from one image. The modified testing set with a size of 48 × 48 pixels was fed into the trained lightweight U-Net. The predicted outcomes of the patches were then placed back to create the integral prediction maps of the original images. The final integral outcomes that the algorithms would present for each image were prediction maps with each pixel indicating the probability of being diagnosed as laser marks, calculated by the sum of the prediction results divided by the frequency of being predicted.

### 2.3. A Lightweight U-Net Model Development

The lightweight U-Net structure was proposed by Wang et al. [24], who used this structure for the segmentation of retinal vessels in single-channel images. Our model resembled the structure of Wang et al., and we applied this method to segment the laser marks of the fundus photographs.

The U-Net structure mainly consisted of two paths: the “mutually inverse” contracting path and the expansive path. On each path, dense convolutional bocks, comprising two convolutional layers followed by rectified linear unit (ReLU) layers, were joined by either downsampling operations (on the contracting path) or upsampling operations (on the expansive path).

And the main change from the U-Net to the lightweight U-Net was that we downscaled the original network into a three-scaled network, meaning that there were only five dense convolutional blocks within the entire structure. We further adjusted hyperparameters in the structure:

1. The number of feature channels at every block has been halved compared to the U-Net
2. The padding strategy was adapted in every convolutional operation for storing the marginal information
3. Dropout layers (with probability = 0.2) were introduced between two successive convolutional layers to prevent overfitting

**Figure 2:**
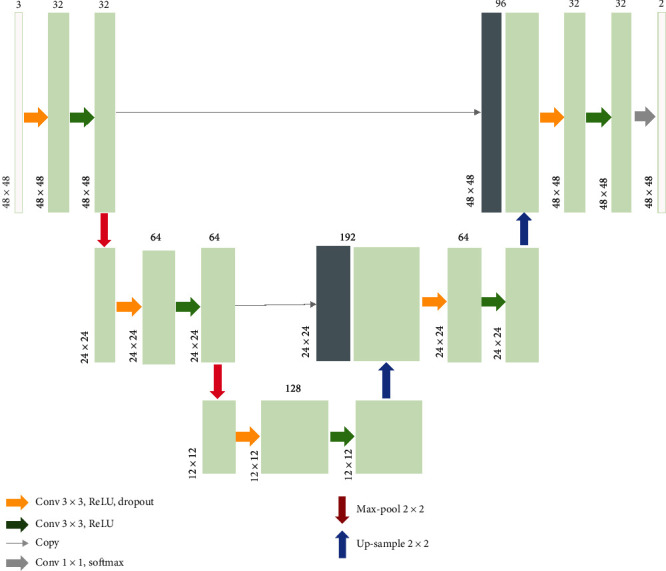
The lightweight U-Net architecture. Each green box corresponded to a multichannel feature map. The number of channels was denoted on top of the boxes. The *x*-*y* size was provided at the lower left edge of the box. Gray boxes represented copied feature maps. The arrows denoted the different operations.

In the structure proposed by Wang et al., they used convolutional filters with a size of 3 × 3 and a striding step of 2 to down-sample the feature maps, but our model simply used the max-pooling operations with filters of size 2 × 2 to downsize the feature maps.  Figure 2 vividly shows the structure of the lightweight U-Net. The detailed parameters of the layers are also presented in Figure 2.

We used the transfer learning technique for parametric initialization before formally training the networks with images containing accurately labeled laser marks. We first pretrained the modified lightweight U-Net with the DRIVE dataset [25], a fundus image dataset with a total of 40 images annotating vessels. Likewise, 40 images in DRIVE went through the data preprocessing, and augmentation procedures mentioned above before being used for pretraining. Although DRIVE was not built for the segmentation of laser marks, using it to pretrain the network did help accelerate the convergence of parameters and obtain more accurate results because both datasets shared similar characteristics [26]. Subsequently, we pretrained the lightweight U-Net with the 84 roughly labeled fundus images. This helped the network to grasp a coarse cognition of laser marks.

Our extended training set was randomly divided into batches, each possessing 128 patches. Ten percent of the samples of a batch was split apart for validation. The stochastic gradient descent (SGD) was applied for parameter optimization by minimizing the loss function. The loss function in this segmentation task was a pixel-wise categorical crossentropy, which computed the following formula over the final feature maps:

(3) $$\begin{array}{c} R=-\overset{N}{\underset{i=1}{\sum }}\overset{K}{\underset{k=1}{\sum }}y_{ik}\log f_{k}\left(x_{i}\right), \end{array}$$

where *N* indicates the total number of pixels within a batch, i.e., *N* = batch size × pixel number per patch, *K* denotes the number of classes, and *y*_*ik*_ and *f*_*k*_(*x*_*i*_) represent the ground truth label and the predicted probability of the *x*_*i*_ pixel in the *k*th class, respectively. Network parameters (weights) saved for testing were the weights that minimized the loss function evaluated in the validation set.

### 2.4. Postprocessing Procedures

PRP surgeries generally follow certain standards, and there are several standards directly related to positioning and screening laser marks on fundus images in clinical practice. Here are two examples:

- Lasers should not be beamed within the OD and a diameter range of the OD
- Lasers should not be beamed within 1500 microns from the macular fovea

We developed two postprocessing procedures to denoise the prediction maps based on these standards. Below is a sketchy description of the algorithms.

As to the first standard, all the “suspected laser marks” at the ODs and their peripheries are not laser marks. Thus, it is necessary to eliminate the false-positive judgments around the ODs. To do this, we first located the ODs and then erased the positive decisions within an elliptical area at and around the ODs. We applied a deep learning method to locate the ODs. The deep learning structure and training process were identical to the lightweight U-Net, except that it was pretrained only on the DRIVE dataset and then trained on the fundus images with ODs manually segmented. This fundus photograph dataset annotating ODs was collected and annotated at the Image Reading Center of Zhongshan Ophthalmic Center. The predicted OD area on each segmentation map was determined as the largest connected domain on the binary prediction graph, where the division threshold of the binary prediction graph from the grayscale output was set to be 0.5 empirically. This method has proven effective in locating and segmenting this anatomic structure in our prior experiment on an inhouse image dataset: evaluating on the inhouse testing set, the area under the receiver operating characteristic curve (AUC) was 0.9997, and the sensitivity, specificity, and accuracy were 93.90%, 99.90%, and 99.81%, respectively. The major and minor axes of elliptical areas that would be covered over the ODs were determined by 1.8 times the maximal *x*-axis and *y*-axis lengths of the predicted OD.

**Figure 3:**
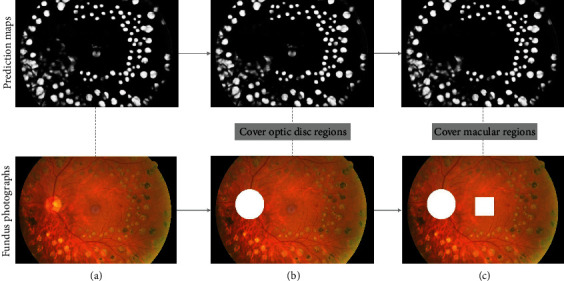
The flowchart of the postprocessing procedures. The top row presented the prediction maps after each step, and the bottom row showed the corresponding procedures on the fundus photographs. (a) The raw prediction map coming from the lightweight U-Net structure and the corresponding fundus image. (b) The prediction map and the fundus image after an elliptic region around the optic disc were covered. (c) The prediction map and the fundus image after the optic disc and macula region were covered.

As to the second standard, what we did was highly analogous. The macular region was first oriented through morphological features using the method proposed by Jiang et al. [27], and then the area around the macular region was covered. To erase more impertinent noises, the area covered was macular-centered squares whose sides were 80 pixels long. Both manipulations served as the backend processes to further optimize the prediction results. The flowchart of the postprocessing is presented in Figure 3.

### 2.5. Hyperparameter Setting for Model Training

The initial learning rate was 1 × 10^−3^ with a learning rate reduce the factor of 0.3 for every ten consecutive epochs without improvement in validation accuracy. The onset of an early stop was when validation accuracy did not improve in 40 consecutive epochs.

### 2.6. Statistical Analysis of Model Performances

Statistical analyses were performed using Python 3.6.5 (Wilmington, Delaware, USA), which was also used for image processing and the lightweight U-Net experiments. Grayscale images were provided as the outcomes of the algorithms, as some shallow shades between bright domains might inform graders of coalesces of laser marks. To evaluate the performances of the lightweight U-Net and its combination with two postprocessing procedures, evaluation metrics were calculated. We drew the receiver operating characteristic (ROC) curves and computed the AUC. Besides, we presented the optimal pairs of sensitivity and specificity on the ROC curves. The optimal choices were based on Youden's index [28], defined as the sum of sensitivity and specificity subtracted by 1, i.e.,

(4) $$\begin{array}{c} \text{Youde}{\text{n}}^{\text{'}}\text{s index}=\text{sensitivity}+\text{specificity}-1. \end{array}$$

Dice similarity coefficient (DSC) can show the percentage of the overlap areas between two set (the prediction map and the manual segmentation map). It equals twice the number of elements in the intersection of both set divided by the sum of the number of elements in each set. The corresponding formula is as follows:

(5) $$\begin{array}{c} \text{ DSC}=\frac{2\text{TP}}{2\text{TP}+\text{FP}+\text{FN}}, \end{array}$$

where *TP*, *FP*, *FN* represent the number of pixels correctly segmented as laser marks (true positive), pixels falsely segmented as laser marks (false positive), and pixels falsely detected as background (false negative), respectively.

## 3. Results

**Figure 4:**
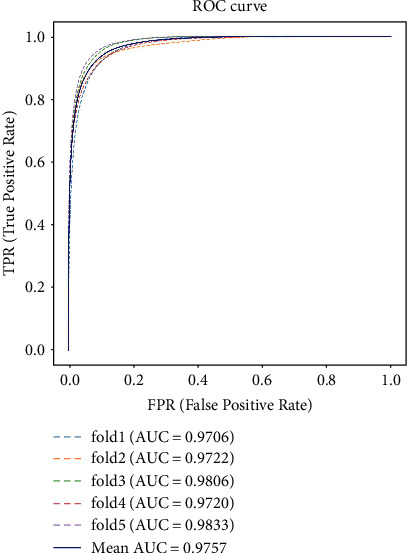
The ROC curves of the 5-fold crossvalidation experiments. The *x*-axis represented the false-positive rate, i.e., 1-specificity, and the *y*-axis represented the true-positive rate, i.e., sensitivity. The area under the curves (AUCs) were calculated, which were presented on the bottom right of the figure. The mean ROC curve, which was plotted with a solid line, and its AUC (namely, mean AUC) were also presented.

Before using the proposed dataset, we did a fivefold crossvalidation to verify the randomness in selecting the training set and the testing set. The 70 elaborately labeled images were randomly divided into five folds, and each fold contained 14 images. Then, one of the five folds was selected to be the testing set, and the other four folds were the training set in the following validation experiment. The ROC curves of the five crossvalidation experiments without postprocessing procedures and the mean ROC curves were presented in Figure 4. The maximum and minimum AUCs were 0.9833 and 0.9706, respectively. The interval formed by these two extreme AUC values covered 0.9798, indicating that the original division of the training set and the testing set were relatively random.

**Figure 5:**
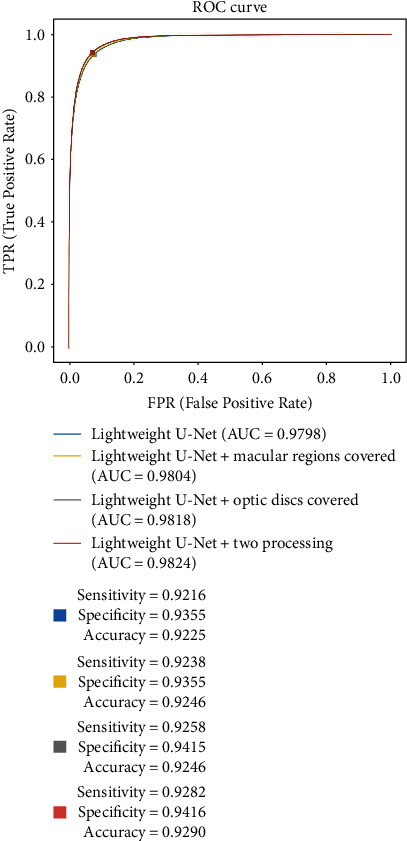
The ROC curves of the lightweight U-Net, the lightweight U-Net with either postprocessing procedure, and with both postprocessing procedures. The *x*-axis represented the false-positive rate, i.e., 1-specificity, and the *y*-axis represented the true-positive rate, i.e., sensitivity. The area under the curve (AUC) was also calculated, which was presented on the bottom right of the figure. The optimal pair of sensitivity and specificity chosen based on Youden's index were also marked on the curve, and the precise numeric values were presented on the right side.

**Figure 6:**
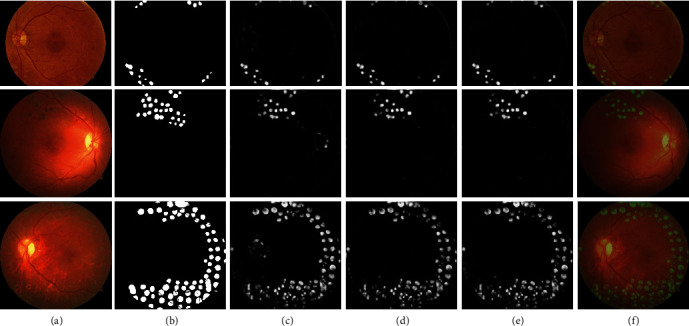
Dataset and prediction result presentation. Each row represented a sample in the testing sets and its corresponding processed results. (a) Laser marks showed in the fundus photographs of patients with DR. (b) The ground truth labels of laser marks in the testing images. (c) The raw predictions for laser marks made by the lightweight U-Net. (d) The predictions for laser marks made by the lightweight U-Net after optic disc areas were covered. (e) The postprocessed lightweight U-Net showed more accurate outcomes in the segmentation of laser makers. (f) The merge of postprocessed prediction maps and the original fundus photographs.

For the model evaluation, ROC curves of predictions through the lightweight U-Net alone and with the postprocessing procedures were presented in Figure 5. The optimal pairs of sensitivity and specificity on the ROC curves and the pixel-wise accuracies corresponding to them were shown in Table 1, which contained the results on a test set trained by lightweight U-Net and the net with postprocessing. The AUC for the 20 testing images was 0.9824 for the lightweight U-Net structure with the postprocessing procedures, which increased the AUC index by 0.26% compared with the AUC for the structure without these procedures. The best sensitivity, specificity, and accuracy for the lightweight U-Net with the postprocessing procedures were 94.16%, 92.82%, and 92.90%, respectively, attaining a, respectively, 0.61%, 0.66%, and 0.65% rise compared to those of the light structure without postprocessing procedures. And the DSC also verified the results that the postprocessing procedures brought in a small advance. The small scale of improvement might result from the low probability of noises around ODs and macular regions. The effect of the postprocessing procedures on the evaluation metrics was not as great as the effect on the display of the segmentation results. Examples of the raw prediction images generated from the lightweight U-Net and the corresponding postprocessed prediction images were shown in Figure 6. After postprocessing procedures, noises at and around the ODs and macular regions were removed, leaving clearer and more accurate segmentation results. Finally, an algorithm according to the definition of PRP for diagnosing was designed, and the prediction results were used to verify in the test set. Through the prediction of laser marks, the sensitivity, specificity, and accuracy of our algorithm are 80%, 100%, and 90%, respectively.

Both the quantitative evaluations and the predicted maps showed that the combination with prior medical knowledge improved the performance of the deep learning algorithm and thus could achieve a good result even with a small amount of data.

## 4. Discussion

To the best of our knowledge, we first developed the lightweight U-Net, a deep learning algorithm used to segment the laser marks in fundus photographs in the present study. Traditional methods also focus on the detection of laser marks from fundus photos [6–8]. On the one hand, some details in the reported methods, such as some key parameters, are not clearly provided in the articles; so, we cannot use the accurate parameters to get good performance results. On the other hand, the segmentation of laser marks by traditional morphological methods is relatively poor and rough, for the morphological methods do not extract the candidate regions exactly. On the contrary, the deep learning method can well learn the essential features of retinal laser lesions by training on a well-labeled dataset and perform better than the morphological methods [29]. The neural network was trained and evaluated on the dataset of novel fundus photographs with laser marks in this study. Moreover, two postprocessing procedures developed from clinical standards of the PRP surgery were combined with the lightweight U-Net to further improve the segmentation results. Both the high numerical metrics and grayscale output images showed the lightweight U-Net could be used to set apart the laser marks on the fundus images, thus providing visible explanations of computer aid systems for DR diagnosis.

The biggest challenge of AI is the interpretability of deep learning algorithms. When a deep learning model detects a fundus photograph as an image with DR, it always gives out the diagnosis without providing more interpretable reasons why it made the decision. In this study, we first adopted a convolutional neural network designed for image segmentation, the lightweight U-Net, to detect laser marks from fundus photographs. This network assigned class labels to every pixel of the images and the pixel-based method to some extent visualized the diagnosis of the R3S stage of DR. The segmentation probability maps provided not only the suspected locations of laser marks but the valuable information for ophthalmologists, who could fully consider the comprehensive conditions of the patients and thus could provide more accurate and suitable cares and adjust the treatment regimens for the patient with DR. The focal or grid laser marks due to diabetic macular edema in our data were relatively lighter, smaller, and less pigment than the laser spots in other parts of the retina. While the experienced graders in our study marked all kinds of laser marks including the focal or grid laser marks accurately, we combined different laser marks images together for the training of the deep learning algorithm. Thus, it would not reduce the accuracy of segmentation in the test set. Combined with the additional algorithm, PRP could be accurately diagnosed according to the segmentation of laser marks, and the diagnostic accuracy of PRP on the test set was 90%, assisting the interpretative diagnosis of R3S. All the laser marks with the different color or size used for segmentation in this study were from the fundus photographs of patients with DR. However, the features of retinal laser marks treated different diseases are similar, and the algorithm in our study could also make accurate predictions for laser markers in the fundus photographs of patients with retinal vein occlusion or peripheral retinal breaks.

Another major challenge in building computer-aided diagnosis systems is that medical knowledge is difficult to merge with deep learning algorithms. Combining prior medical knowledge undoubtedly improves the decision accuracy of a deep learning model. The present work addressed this challenge by collaborating the results of multimodels, which was to postprocess the segmentation results of the lightweight U-Net. According to clinical practices that the areas around OD and macular regions do not allow the presence of laser marks, we applied a similar deep network structure to cover the elliptic area containing the OD and a classical morphological operation to locate macular regions and then denoised around them. These two procedures were rooted in clinical practices and made the results closer to reality. The coverage of ODs and their peripheries improved the lightweight U-Net results more than the coverage of macular regions, which might be because ODs and laser marks shared more morphological characteristics, resulting in more misclassification around the optic disc areas for the lightweight U-Net model. The combination with the postprocessing procedures did not require graders a great effort to label a large amount of laser mark. With a relatively small amount of data for training, along with the cooperation with other models, the refined results containing a priori medical experience could be obtained. The postprocessing procedures derived from the clinical practices in this study increased the efficiency of the lightweight U-Net. The result concatenation from multiple models in our study was a simple but effective way to incorporate medical knowledge.

There were still several limitations in our study. First, our model showed its high reliability in distinguishing most laser marks from retinas, but it was still hard to differentiate some noises sharing similar morphological characteristics. Therefore, we will try to adjust the image tone in preprocessing and coalesce local information with global information in the future. Second, the number of fundus photographs in the training set was still relatively small. With more training images from diverse populations, the model would be more robust and accurate.

## 5. Conclusions

This study developed the postprocessed lightweight U-Net to accurately and reliably segment laser marks in fundus photographs for the AI-assisted diagnosis of DR in the different stage, potentially reducing the workload of oculists in various fundus diseases to some extent in the near future.

## Figures and Tables

**Table 1:** Comparison of methods on the proposed dataset.

Architecture	Sensitivity $=\frac{TP^{*}}{\text{TP}+\text{F}{\text{N}}^{†}}$	Specificity $=\frac{\text{T}{\text{N}}^{‡}}{\text{TN}+\text{F}{\text{P}}^{§}}$	Accuracy $=\frac{\text{TP}+\text{TN}}{\text{TP}+\text{FP}+\text{TN}+\text{FN}}$	DSC	AUC
Lightweight U-Net	93.55%	92.16%	92.25%	70.59%	0.9798
Lightweight U-Net with macular regions covered	93.55%	92.38%	92.46%	70.76%	0.9804
Light weight U-Net with optic discs covered	94.15%	92.58%	92.68%	71.01%	0.9818
Lightweight U-Net with both post-processing procedures	94.16%	92.82%	92.90%	71.18%	0.9824

^∗^TP: the number of pixels correctly classified as laser marks; †FN: the number of pixels mistakenly classified as background; ‡ TN: the number of pixels correctly classified as background; § FP: the number of pixels mistakenly classified as laser marks.

## Data Availability

Original data and corresponding labels of training and test set are publicly available as attachments.
